# Traditional Machine Learning, Deep Learning, and BERT (Large Language Model) Approaches for Predicting Hospitalizations From Nurse Triage Notes: Comparative Evaluation of Resource Management

**DOI:** 10.2196/52190

**Published:** 2024-08-27

**Authors:** Dhavalkumar Patel, Prem Timsina, Larisa Gorenstein, Benjamin S Glicksberg, Ganesh Raut, Satya Narayan Cheetirala, Fabio Santana, Jules Tamegue, Arash Kia, Eyal Zimlichman, Matthew A Levin, Robert Freeman, Eyal Klang

**Affiliations:** 1 Institute for Healthcare Delivery Science Icahn School of Medicine at Mount Sinai New York, NY United States; 2 Division of Diagnostic Imaging Sheba Medical Center Tel-Aviv University Tel Aviv Israel; 3 Division of Data-Driven and Digital Medicine (D3M) Icahn School of Medicine at Mount Sinai New York, NY United States; 4 Hospital Management Sheba Medical Center Tel-Aviv University Tel Aviv Israel; 5 ARC Innovation Center Sheba Medical Center Tel-Aviv University Tel Aviv Israel; 6 Department of Anesthesiology, Perioperative and Pain Medicine Icahn School of Medicine at Mount Sinai New York, NY United States; 7 Department of Genetics and Genomic Sciences Icahn School of Medicine at Mount Sinai New York, NY United States; 8 Windreich Department of Artificial Intelligence and Human Health Icahn School of Medicine at Mount Sinai New York, NY United States

**Keywords:** Bio-Clinical-BERT, term frequency–inverse document frequency, TF-IDF, health informatics, patient care, hospital resource management, care, resource management, management, language model, machine learning, hospitalization, deep learning, logistic regression, retrospective analysis, training, large language model

## Abstract

**Background:**

Predicting hospitalization from nurse triage notes has the potential to augment care. However, there needs to be careful considerations for which models to choose for this goal. Specifically, health systems will have varying degrees of computational infrastructure available and budget constraints.

**Objective:**

To this end, we compared the performance of the deep learning, Bidirectional Encoder Representations from Transformers (BERT)–based model, Bio-Clinical-BERT, with a bag-of-words (BOW) logistic regression (LR) model incorporating term frequency–inverse document frequency (TF-IDF). These choices represent different levels of computational requirements.

**Methods:**

A retrospective analysis was conducted using data from 1,391,988 patients who visited emergency departments in the Mount Sinai Health System spanning from 2017 to 2022. The models were trained on 4 hospitals’ data and externally validated on a fifth hospital’s data.

**Results:**

The Bio-Clinical-BERT model achieved higher areas under the receiver operating characteristic curve (0.82, 0.84, and 0.85) compared to the BOW-LR-TF-IDF model (0.81, 0.83, and 0.84) across training sets of 10,000; 100,000; and ~1,000,000 patients, respectively. Notably, both models proved effective at using triage notes for prediction, despite the modest performance gap.

**Conclusions:**

Our findings suggest that simpler machine learning models such as BOW-LR-TF-IDF could serve adequately in resource-limited settings. Given the potential implications for patient care and hospital resource management, further exploration of alternative models and techniques is warranted to enhance predictive performance in this critical domain.

**International Registered Report Identifier (IRRID):**

RR2-10.1101/2023.08.07.23293699

## Introduction

Efficient and effective patient triage within the emergency department (ED) plays a pivotal role in enhancing treatment outcomes and optimizing care delivery [1-3]. This process involves rapidly identifying patients who require immediate hospitalization upon their arrival. One of the resources for making these predictions are nurse triage notes, which provide a wealth of in-depth information about the patient’s condition at presentation [4,5].

In the field of health care, machine learning has opened up new avenues for potential improvement in such complex classification tasks, thereby augmenting clinical decision-making processes [6,7]. The recent developments in deep learning and natural language processing (NLP) techniques have further broadened this potential, bringing a new realm of possibilities for enhancing medical decision-making capabilities.

Among these advanced algorithms is the Bidirectional Encoder Representations from Transformers (BERT) model [8]. BERT has shown excellent performance in numerous NLP tasks [9] and has inspired the development of more specialized versions tailored to particular fields, such as the Bio-Clinical-BERT model, which was designed to cater to the biomedical field [10].

The focus of this study is to delve into the potential of a fine-tuned Bio-Clinical-BERT model and compare it against a simpler, robust, and more traditional approach, mainly, the bag-of-words (BOW) logistic regression (LR) model complemented by the term frequency–inverse document frequency (TF-IDF) method. We also evaluated other approaches including the extreme gradient boosting (XGBoost) classifier and Word-2-Vec (W2V) embedding with bidirectional long short-term memory (Bi-LSTM) network. The primary objective of our research is to gauge the efficacy of these 2 methods in predicting hospital admissions using nurse triage notes.

While it is true that Bio-Clinical-BERT could potentially offer improved accuracy in its predictions, it should be noted that it also requires a substantial investment in terms of computational resources. It necessitates the use of specialized hardware and demands a certain level of software expertise to operate effectively. On the other hand, the LR model paired with the TF-IDF method is more resource efficient and enjoys wide acceptance in the field of text classification due to its simplicity and effectiveness.

We hypothesized that the Bio-Clinical-BERT model may surpass the performance of the BOW-LR model combined with the TF-IDF approach in the task of predicting triage outcomes. However, we also speculated that the incremental gains in performance might not necessarily justify the additional demands imposed by the large deep learning model in terms of computational resources and technical know-how. To test this hypothesis, we have undertaken an extensive study using over 1 million nurse triage notes collected from a large health system.

The fundamental contribution of this paper is a comparison between these techniques for predicting hospital admission, which reflect different levels of computational requirements and cost implications. Our comparison not only looks at the accuracy of these models but also weighs the trade-offs between predictive accuracy and computational efficiency, a consideration that is often overlooked but is of prime importance in real-world settings when implementing models. Specifically, health systems may be able to use insights from this study to make informed decisions on which methodology may be right for their circumstances, with a clearer understanding of the limitations of each. Our aim is to equip health care practitioners, researchers, and decision makers with insights that could potentially aid in enhancing hospital resource management and improve the quality of patient care.

## Methods

### Data Sources and Study Design

For the construction and testing of our models, we used an extensive dataset from the Mount Sinai Health System (MSHS). This is a diverse health care provider based in New York City. In this study, the dataset included ED records spanning a 5-year period from 2017 to 2022. This dataset was gathered from 5 different MSHS hospitals, covering a broad range of population groups and diverse urban health settings.

These 5 participating hospitals provided a rich source of data for our study, representing different communities in New York City. The hospitals include Mount Sinai Hospital, a health care institution located in East Harlem, Manhattan; Mount Sinai Morningside, situated in Morningside Heights, Manhattan; Mount Sinai West, operating in Midtown West, Manhattan; Mount Sinai Brooklyn, a community-focused health facility located in Midwood, Brooklyn; and Mount Sinai Queens (MSQ), based in Astoria, Queens. The dataset used for our study was compiled using the Epic Electronic Health Records software, a tool that aids in efficient data collection, management, and analysis. The dataset was made available by the diligent work of the Mount Sinai Hospital Clinical Data Science team.

### Model Development and Evaluation

In the development and testing of our models, we leveraged data from 4 hospitals for training, validation, and hyperparameter tuning processes. We elected to use a distinct dataset from MSQ for external testing to ensure our model’s generalizability.

The internal training and validation cohort underwent a procedure involving 5-fold cross-validation. Each fold contained 10,000 records, which were used for hyperparameter tuning. For the external dataset, we experimented with training sets of varying sizes: 10,000; 100,000; and roughly 1,000,000 patients, which represent the complete 4-hospital cohort. Subsequently, testing was carried out on 20% of these cohorts’ sizes, taken from the MSQ hospital cohort.

Our study involved several models: Bio-Clinical-BERT and BOW-LR models using TF-IDF features. For further subanalyses using different machine and deep learning models, we also evaluated XGBoost with BOW and Bi-LSTM with a W2V pretrained embedding layer derived from bioclinical data (BioWordVec_PubMed_MIMICIII_d200).

As a final subanalysis experiment, for the BERT model, we also experimented with up-sampling of the minority class to ensure balanced data representation, enhancing the stability and accuracy of our model predictions.

These models were used to predict hospitalization outcomes from nurse triage notes. For Bio-Clinical-BERT, we adhered to text preprocessing and tokenization guidelines as outlined on the Hugging Face website [11].

For BOW-XGBoost, we evaluated 3 different numbers of estimators. Other XGBoost hyperparameters were set to default values, including a learning rate of 0.3, maximum depth of 6, and minimum child weight of 1.

For W2V-Bi-LSTM, the network is comprised of a Bi-LSTM layer (256 hidden units), preceded by a pretrained embedding 200-dimensions W2V layer, with a fully connected layer followed by a sigmoid activation function.

Further details on hyperparameter selection are elucidated in the *Hyperparameter Tuning Results* section. For BOW-LR-TF-IDF, we followed a similar methodology outlined in our previous publication [12], covering both text preprocessing and hyperparameter selection processes.

BERT is a model designed for NLP tasks. It learns from the context of both preceding and following words, making it “bidirectional.” This model is pretrained on large corpora and can be fine-tuned for specific tasks.

The BOW model is a simple technique in NLP. It represents text data by counting the frequency of each word, disregarding the order in which they appear. Each unique word forms a feature, and the frequency of the word represents the value of that feature. However, this method can overlook context and semantics due to its simplicity.

TF-IDF is a numerical statistic that reflects how important a word is to a document in a collection. It is a combination of 2 metrics: *term frequency*, which is the number of times a word appears in a document, and *inverse document frequency*, which diminishes the weight of common words and amplifies the weight of rare words across the entire dataset. This helps in reducing the impact of frequently used words and highlights more meaningful terms.

XGBoost is an advanced gradient boosting framework known for its efficiency and performance in structured data classification and regression. It builds multiple decision trees sequentially to correct previous errors, excelling in handling diverse data types and preventing overfitting.

Bi-LSTM is an artificial neural network that processes data in both directions to capture past and future context. This enhances its sequence understanding, making it suitable for text classification, sentiment analysis, and machine translation.

### Study Population

The demographic for this study included adult patients aged 18 years and older. These were patients who made ED visits within the specified 5-year period from 2017 to 2022 across the 5 participating MSHS hospitals.

### Outcome Definition

The primary outcome for our study was to ascertain our models’ effectiveness in predicting hospitalization. This prediction was based on 2 main types of data: tabular electronic health records and nurse triage notes.

### Model Evaluation and Comparison

To assess the performance of our models, we used various metrics such as area under the receiver operating characteristic curve (AUC), sensitivity, specificity, and precision. These metrics allowed us to thoroughly evaluate the Bio-Clinical-BERT [10] and BOW-LR models with TF-IDF features, as well as compare their capabilities in predicting hospitalization from nurse triage notes.

### Ethical Considerations

This study, being retrospective in nature, was reviewed and approved by an ethical institutional review board committee from the MSHS (protocol: STUDY-18-00573). The institutional review board committee deemed that due to the retrospective nature of the study, the requirement for informed consent was waived.

### Statistical Analysis

Our statistical analyses were conducted using Python (version 3.9.12; Python Software Foundation). We presented continuous variables as median (IQR) and categorical variables as percentages for better interpretability. To identify words linked to hospital admission within nurse triage notes, we calculated the odds ratio (OR) and mutual information (MI) [12]. Statistical tests such as the chi-square test and 2-tailed Student *t* test were used for comparing categorical and continuous variables, respectively. A *P* value <.05 was considered statistically significant. For evaluating our models, receiver operating characteristic (ROC) curves were plotted, and metrics including AUC, sensitivity (recall), specificity, and positive predictive value (precision) were derived.

### Technical Architecture

**Figure 1:**
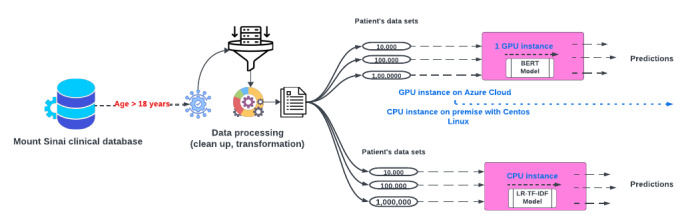
Process flow of multiple patient datasets passing through 2 different models with GPU and non-GPU instances. BERT: Bidirectional Encoder Representations from Transformers; LR: logistic regression; TF-IDF: term frequency–inverse document frequency.

The technical experiments involved in this study were conducted within a controlled hospital infrastructure that used an On-Premises Centos Linux environment in conjunction with Azure Cloud infrastructure. For the BOW-TF-IDF experiments, we elected to use the Centos Linux OS. In contrast, the BERT experiment was conducted using a Standard_NC6 GPU instance on Azure Cloud. This instance came with one 16-GB GPU and 6 vCPUs and incurred a cost of approximately US $80 during the training phase. Figure 1 offers a detailed depiction of the fundamental technical architecture used for training the BERT and LR-TF-IDF models, across multiple patient datasets.

## Results

### Patient Population and Data

Our study incorporated data from 1,745,199 patients drawn from the MSHS. Upon the exclusion of patients aged <18 years, we had 1,391,988 participants in the study. These patients visited the ED between 2017 and 2022. Table 1 presents a summary of the patient characteristics.

The median number of words per triage note was 19.0 (IQR 12.0-31.0). Top 10 words associated with the highest MI score regarding hospital admission are outlined in Table 2.

**Table 1:** Demographic distribution in the study.

Demographics		All patients (includes MSH^a^, MSM^b^, MSW^c^, MSB^d^, and MSQ^e^; N=1,391,988)	4 hospitals (MSH, MSM, MSW, and MSB; n=1,110,272)	MSQ (n=281,716)	*P* value	
Age (years), median (IQR)		47.0 (31.0-75)	48.0 (32.0-75.0)	45.0 (30.0-75.0)	<.001	
**Sex, n (%)**						<.001
	Female	727,363 (52.3)	586,224 (52.8)	141,140 (50.1)		
	Male	664,625 (47.7)	524,048 (47.2)	140,576 (49.9)		
**Race, n (%)**						<.001
	Black	428,594 (30.79)	382,898 (34.5)	45,696 (16.22)		
	White	343,079 (24,65)	265,457 (23.92)	77,622 (27.56)		
	Other	620,315 (44,56)	461,917 (41.58)	158,398 (56.22)		

^a^MSH: Mount Sinai Hospital.

^b^MSM: Mount Sinai Morningside.

^c^MSW: Mount Sinai West.

^d^MSB: Mount Sinai Brooklyn.

^e^MSQ: Mount Sinai Queens.

**Table 2:** Odds ratios (OR) and mutual information (MI) values for words linked to admission to hospital wards, sorted by highest MI values.

Word	OR for admission	MI for admission	*P* value
Sent	3.6	16.4	<.001
Pt^a^	1.6	15.8	<.001
Per	2.3	15	<.001
Of	1.3	12.7	<.001
Home	2.2	11.5	<.001
EMS^b^	2.2	10.8	<.001
Weakness	3.6	10.8	<.001
Chest	1.4	8.9	<.001
SOB^c^	2.1	8.8	<.001
BIBA^d^	2.1	7.9	<.001

^a^Pt: patient.

^b^EMS: emergency medical services.

^c^SOB: shortness of breath.

^d^BIBA: brought in by ambulance.

### Hyperparameter Tuning Results

A hyperparameter tuning process was performed. The best hyperparameters were identified for each model based on their performance during the 5-fold cross-validation on the training validation set. The results of the BERT hyperparameter tuning process can be found in Table 3.

The results of the W2V-LSTM model hyperparameter tuning are presented in Table 4.

The results of XGBoost hyperparameter tuning are presented in Table 5.

**Table 3:** BERT^a^ hyperparameter tuning in the internal training and validation cohorts using 5-fold experiments.

Batch size	Max length	Learning rate	Epoch	Value, mean (SD)
64	—^b^	2×10^–5^	—	0.78 (0.01)
128	—	2×10^–5^	—	0.80 (0.01)
128	128	2×10^–5^	3	0.80 (0.01)
256	64	2×10^–5^	—	0.79 (0.01)
64	—	3×10^–5^	—	0.79 (0.01)
128	—	3×10^–5^	—	0.79 (0.01)
128	128	3×10^–5^	3	0.78 (0.01)
256	64	3×10^–5^	—	0.78 (0.01)
64	—	5×10^–5^	—	0.79 (0.01)
128	—	5×10^–5^	—	0.80 (0.01)
128	128	5×10^–5^	3	0.79 (0.01)
256	64	5×10^–5^	—	0.79 (0.01)

^a^BERT: Bidirectional Encoder Representations from Transformers.

^b^Not applicable.

**Table 4:** W2V^a^-LSTM^b^ hyperparameter tuning in the internal training and validation cohorts using 5-fold experiments.

Batch size	Learning rate	Epochs	AUC^c^, mean
16	10^−3^	5	0.768
16	10^−3^	10	0.795
16	10^−3^	15	0.765
16	10^−4^	5	0.750
16	10^−4^	10	0.781
16	10^−4^	15	0.798
32	10^−3^	5	0.797
32	10^−3^	10	0.797
32	10^−3^	15	0.777
32	10^−4^	5	0.756
32	10^−4^	10	0.728
32	10^−4^	15	0.748
64	10^−3^	5	0.661
64	10^−3^	10	0.806
64	10^−3^	15	0.795
64	10^−4^	5	0.693
64	10^−4^	10	0.767
64	10^−4^	15	0.775

^a^W2V: Word-2-Vec.

^b^LSTM: long short-term memory.

^c^AUC: area under the receiver operating characteristic curve.

**Table 5:** XGBoost^a^ hyperparameter tuning in the internal training and validation cohorts using 5-fold experiments.

Trees	Value
100	0.80 (0.01)
200	0.81 (0.01)
1000	0.80 (0.01)

^a^XGBoost: extreme gradient boosting.

### Model Performance

After training the Bio-Clinical-BERT and LR-TF-IDF models on the 4 hospitals’ data, we evaluated their performance on the held-out test data from MSQ. The AUC values were calculated for each model. The Bio-Clinical-BERT model achieved AUCs of 0.82, 0.84, 0.85, while the LR-TF-IDF model had AUCs of 0.81, 0.83, 0.84 for training on 10,000; 100,000; and ~1,000,000 patients, respectively.

**Figure 2:**
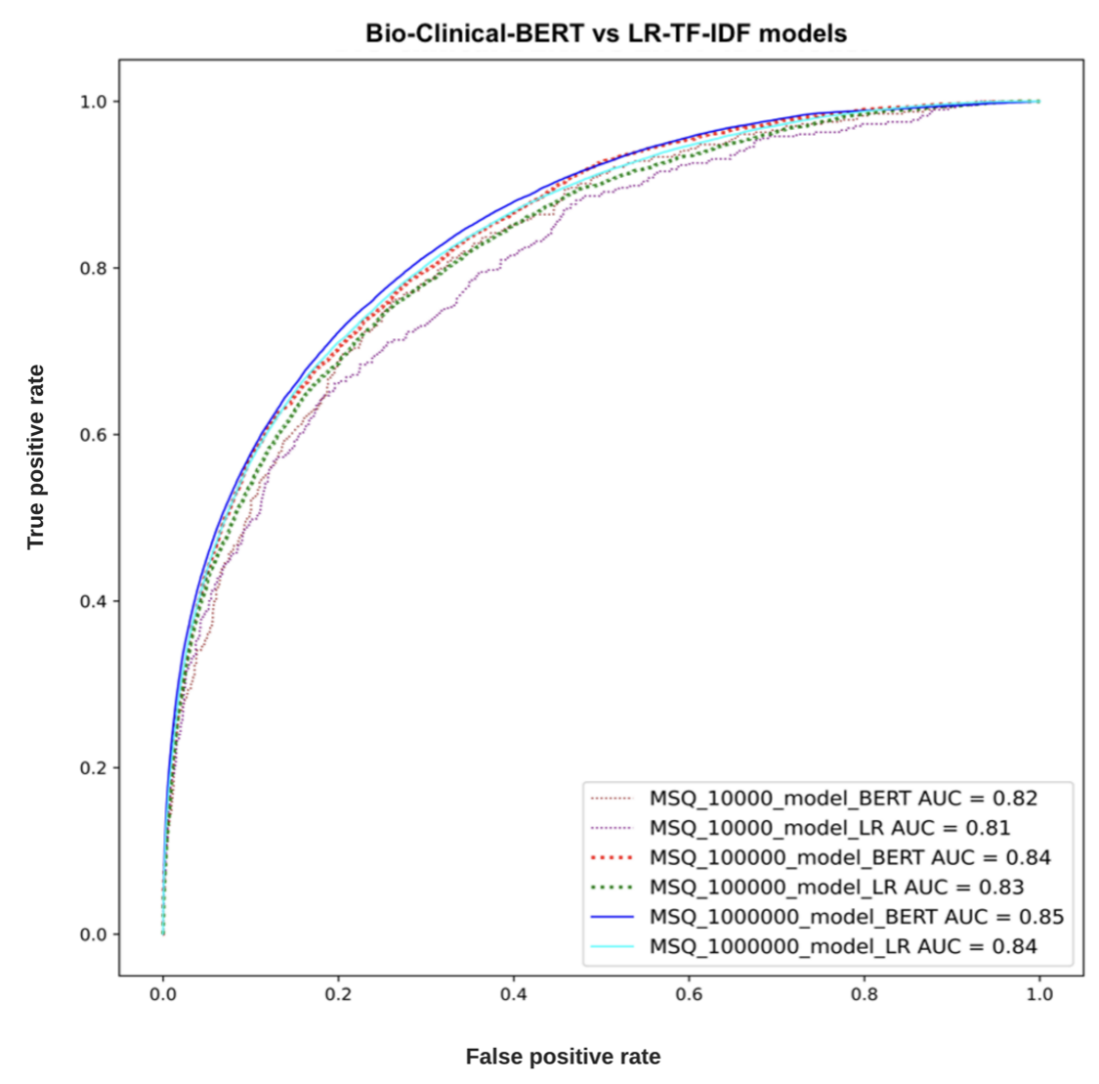
Receiver operating characteristic curves (ROC) of the 2 models tested on increasing training sample sizes. AUC: area under the receiver operating characteristic curve; BERT: Bidirectional Encoder Representations from Transformers; LR: logistic regression; MSQ: Mount Sinai Queens; TF-IDF: term frequency–inverse document frequency.

Figure 2 shows the ROC and AUC comparisons between the 2 models. The Bio-Clinical-BERT model consistently outperformed the LR-TF-IDF model in terms of AUC across the different training set sizes (10,000; 100,000; and ~1,000,000 patients), albeit by a small margin.

In addition to the AUC comparisons, we also calculated other performance metrics, such as sensitivity, specificity, and precision, for both models (Table 6 and Table 7).

**Table 6:** Metrics for the training and testing (external) cohorts for the Bio-Clinical-BERT^a^ model.

Training data size	AUC^b^ score	Sensitivity	Specificity	Precision	*F*_1_-score
10,000	0.82	0.76	0.74	0.36	0.49
100,000	0.84	0.74	0.77	0.39	0.51
1,000,000	0.85	0.39	0.96	0.67	0.50

^a^BERT: Bidirectional Encoder Representations from Transformers.

^b^AUC: area under the receiver operating characteristic curve.

**Table 7:** Metrics for the training and testing (external) cohorts for the LR^a^-TF-IDF^b^ model.

Training Data Size	AUC^c^ score	Sensitivity	Specificity	Precision	*F*_1_-score
10,000	0.81	0.66	0.80	0.40	0.50
100,000	0.83	0.75	0.74	0.37	0.50
1,000,000	0.84	0.71	0.80	0.42	0.53

^a^LR: logistic regression.

^b^TF-IDF: term frequency–inverse document frequency.

^c^AUC: area under the receiver operating characteristic curve.

The metrics for the XGBoost and W2V-Bi-LSTM models are presented in Tables 8 and 9. The probability cutoff values for these metrics were calculated using the Youden index. These results further demonstrated the superior performance of the Bio-Clinical-BERT model compared to the LR-TF-IDF model.

Further subanalysis for the BERT cohort using up-sampling of the minority class is presented in Table 10.

**Table 8:** Metrics for the training and testing (external) cohorts for the W2V^a^-Bi-LSTM^b^ model.

Training data size	AUC^c^ score	Sensitivity	Specificity	Precision	*F*_1_-score
10,000	0.78	0.32	0.95	0.59	0.41
100,000	0.81	0.42	0.92	0.52	0.46
1,000,000	0.84	0.46	0.94	0.62	0.52

^c^W2V: Word-2-Vec.

^c^Bi-LSTM: bidirectional long short-term memory.

^c^AUC: area under the receiver operating characteristic curve.

**Table 9:** Metrics for the training and testing (external) cohorts for the XGBoost^a^ model.

Training data size	AUC^b^ score	Sensitivity	Specificity	Precision	*F*_1_-score
10,000	0.76	0.21	0.97	0.69	0.33
100,000	0.81	0.27	0.98	0.73	0.39
1,000,000	0.82	0.33	0.97	0.69	0.45

^a^XGBoost: extreme gradient boosting.

^b^AUC: area under the receiver operating characteristic curve.

**Table 10:** Metrics for the training and testing (external) cohorts for BERT^a^ with up-sampling of the minority class.

Training data size	AUC^b^ score	Sensitivity	Specificity	Precision	*F*_1_-score
10,000	0.79	0.81	0.58	0.30	0.43
100,000	0.84	0.81	0.68	0.34	0.48
1,000,000	0.85	0.75	0.78	0.41	0.54

^a^BERT: Bidirectional Encoder Representations from Transformers.

^b^AUC: area under the receiver operating characteristic curve.

## Discussion

In this study, we compared the performance of several predictive models, including Bio-Clinical-BERT and LR-TF-IDF, in predicting hospitalizations based on nurse triage notes. The findings of our study suggest that while Bio-Clinical-BERT does marginally outperform LR-TF-IDF in this predictive task, the difference in their performance is relatively minor.

Such results echo the findings of previous studies in the field, which have often found BERT-based models to have a slight edge over more traditional methods such as LR-TF-IDF in various NLP tasks [13,14]. However, the marginal difference observed in our study suggests that, given certain limitations such as constraints on hardware, software expertise, or budget, hospitals might lean toward simpler methods. The rationale behind such a choice would lie in the ease of implementing these simpler methods, as well as their relatively less demanding computational requirements.

The comparison of different models in the biomedical domain has been the focus of numerous previous studies. For instance, Chen et al [15] conducted an assessment of transformer-based ChatGPT models in tasks such as reasoning and classification. Their study found that fine-tuning remained the most effective approach for 2 central NLP tasks. However, it is interesting to note that the basic BOW model managed to deliver comparable results to the more complex language model prompting. It should be noted that the creation of effective prompts required a substantial resource investment.

In another study, Xavier and Chen [16] compared 3 different model types for a multiclass text classification task, which involved the assignment of protocols for abdominal imaging computed tomography scans. These models spanned a range from conventional machine learning and deep learning to automated machine learning builder workflows. While the automated machine learning builder boasted the best performance with an *F*_1_-score of 0.85 on an unbalanced dataset, the tree ensemble machine learning algorithm was superior on a balanced dataset, delivering an *F*_1_-score of 0.80.

A further study delved into the evaluation of machine learning multiclass classification algorithms’ performance in classifying proximal humeral fractures using radiology text data [17]. Several statistical machine learning algorithms were performed, with a BERT model showcasing the best accuracy of 61%. In another relevant study conducted by Ji et al [18], various models pretrained with BERT were compared for medical code assignment based on clinical notes. Interestingly, it was found that simpler artificial neural networks could sometimes outperform BERT in certain scenarios. This study, among others, offers further support to our recommendation for hospitals with limited resources to consider simpler, less resource-demanding methods for achieving comparable predictive performance.

In the specific task of predicting hospitalization, both methods in our study effectively leveraged the rich information found within nurse triage notes. This finding aligns with those from other studies [19-21]. For instance, a study by Zhang et al [19] that evaluated LR and neural network modeling approaches in predicting hospital admission or transfer after initial ED triage presentation found that the patient’s free-text data regarding referral improved overall predictive accuracy. Similarly, Raita et al [20] used machine learning models to predict ED outcomes and demonstrated superior performance in predicting hospitalization.

The results of our study carry practical implications for health care organizations. The ability to predict hospitalization from nurse triage notes could lead to improvements in patient care by facilitating efficient resource allocation, optimizing bed management, and improving patient flow.

The choice between the use of Bio-Clinical-BERT and simpler methods, such as LR-TF-IDF, should be influenced by the specific context of the organization, including factors such as available computational resources, software expertise, and desired model performance.

Our study is not without limitations. For instance, the data used for our study are specific to MSHS hospitals, which might not be representative of other health care systems, potentially limiting the generalizability of our findings. Despite using multisite data, representing the diverse New York City population, and an external validation site for our final analysis, we acknowledge the need for further studies with more diverse datasets, including those that are open source such as the Medical Information Mart for Intensive Care (MIMIC) dataset. We also recognize that we did not explore the potential of combining both methods and other potential techniques that could enhance these models’ performance. The BOW technique by nature does not consider context, which could have hindered performance. There is the possibility that more advanced deep learning models could have achieved a bigger difference in AUC performance compared to the shallow model. Moreover, the field of NLP is advancing fast, and some methodologies were not explored. Also, our study focused on comparative analysis using the Youden index, which may have caused several metrics to be lower than previous publications, such as the *F*_1_-score. Despite this, the models demonstrated high specificity, suggesting potential for clinical use. Further exploration of thresholding methods is necessary to enhance model applicability and performance in real-world settings.

Future research could focus on the exploration of BERT models that are pretrained and trained from scratch on a site’s entire textual data. Although such an approach may demand significant resources and be computationally intensive, it might yield better performance by capturing the unique characteristics and language patterns of a specific health care setting. The exploration of other pretrained language models or more advanced natural language processing techniques could also pave the way for the development of more effective hospitalization prediction methods based on nurse triage notes.

In conclusion, our study demonstrates that while the Bio-Clinical-BERT model does marginally outperform the LR-TF-IDF model in predicting hospitalization from nurse triage notes, the difference is small enough to suggest that simpler methods might be viable for hospitals with limited resources. More research is needed to identify alternative methods that can enhance these models’ performance in predicting hospitalization, ultimately improving patient care and hospital resource management.

Through an investigation of the Bio-Clinical-BERT and LR-TF-IDF models’ performance, our study contributes to the growing body of literature in the field of NLP and machine learning in health care. It emphasizes the importance of considering the trade-offs between model complexity and performance when deploying predictive tools in clinical settings, highlighting that sometimes, simpler methods can prove as effective as more complex ones.

## Abbreviations

AUC: area under the receiver operating characteristic curve
BERT: Bidirectional Encoder Representations from Transformers
Bi-LSTM: bidirectional long short-term memory
BOW: bag-of-words
ED: emergency department
LR: logistic regression
MI: mutual information
MIMIC: Medical Information Mart for Intensive Care
MSHS: Mount Sinai Health System
MSQ: Mount Sinai Queens
NLP: natural language processing
OR: odds ratio
ROC: receiver operating characteristic
TF-IDF: term frequency–inverse document frequency
W2V: Word-2-Vec
XGBoost: extreme gradient boosting
